# Hypermethylation of the CTRP9 promoter region promotes Hcy induced VSMC lipid deposition and foam cell formation via negatively regulating ER stress

**DOI:** 10.1038/s41598-023-46981-5

**Published:** 2023-11-09

**Authors:** Xiuyu Wang, Xing Ma, Yue Zeng, Lingbo Xu, Minghao Zhang

**Affiliations:** 1 Department of Pathophysiology, School of Basic Medical Sciences, Ningxia Medical University, 1160 Shengli Street, Yinchuan, 750004 Ningxia People’s Republic of China; 2 Ningxia Key Laboratory of Vascular Injury and Repair Research, Key Laboratory of Metabolic Cardiovascular Diseases Research of National Health Commission, Yinchuan, 750004 Ningxia People’s Republic of China

**Keywords:** Diseases, Medical research, Pathogenesis

## Abstract

To provide a theoretical basis for the prevention and treatment of atherosclerosis (As), the current study aimed to investigate the mechanism underlying the effect of homocysteine (Hcy) on inducing the lipid deposition and foam cell formation of the vascular smooth muscle cell (VSMC) via C1q/Tumor necrosis factor-related protein9 (CTRP9) promoter region Hypermethylation negative regulating endoplasmic reticulum stress (ERs). Therefore, apolipoprotein E deficient (ApoE^−/−^) mice were randomly divided into the control [ApoE^−/−^ + normal diet (NC)] and high methionine [ApoE^−/−^ + (normal diet supplemented with 1.7% methionine (HMD)] groups (n = 6 mice/group). Following feeding for 15 weeks, the serum levels of Homocysteine (Hcy), total cholesterol (TC), and triglyceride (TG) were measured using an automatic biochemical analyzer. HE and oil red O staining were performed on the aorta roots to observe the pathological changes. Additionally, immunofluorescence staining was performed to detect the protein expression levels of CTRP9, glucose-regulated protein 78 kD (GRP78), phosphorylated protein kinase RNA-like ER kinase (p-PERK), activating transcription factor 6a (ATF6a), phosphorylated inositol-requiring enzyme-1α (p-IRE1α), sterol regulatory element binding proteins-1c (SREBP1c) and sterol regulatory element binding proteins-2 (SREBP2) in VSMC derived from murine aortic roots. In vitro, VSMC was stimulated with 100 μmol/l Hcy. After transfection of plasmids with overexpression and interference of CTRP9, ERs agonist (TM) and inhibitor (4-PBA) were given to stimulate VSMC cells. HE staining and oil red O staining were used to observe the effect of Hcy stimulation on lipid deposition in VSMC. Additionally, The mRNA and protein expression levels of CTRP9, GRP78, PERK, ATF6a, IRE1α, SREBP1c, and SREBP2 in VSMC were detected by RT-qPCR and western blot analysis, respectively. Finally, The methylation modification of the CTRP9 promoter region has been studied. The NCBI database was used to search the promoter region of the CTRP9 gene, and CpG Island was used to predict the methylation site. After Hcy stimulation of VSMC, overexpression of DNMT1, and intervention with 5-Azc, assess the methylation level of the CTRP9 promoter through bisulfite sequencing PCR (BSP). The results showed that the serum levels of Hcy, TC, and TG in the ApoE^−/−^ + HMD group were significantly increased compared with the ApoE^−/−^ + NC group. In addition, HE staining and oil red O staining showed obvious AS plaque formation in the vessel wall, and a large amount of fat deposition in VSMC, thus indicating that the hyperhomocysteinemia As an animal model was successfully established. Furthermore, CTRP9 were downregulated, while GRP78, p-PERK, ATF6a, p-IRE1α, SREBP1c, SREBP2 was upregulated in aortic VSMC in the ApoE^−/−^ + HMD group. Consistent with the in vivo results, Hcy can inhibit the expression of CTRP9 in VSMC and induce ERs and lipid deposition in VSMC. Meanwhile, the increased expression of CTRP9 can reduce ERs and protect the lipid deposition in Hcy induced VSMC. Furthermore, ERs can promote Hcy induced VSMC lipid deposition, inhibition of ERs can reduce Hcy induced VSMC lipid deposition, and CTRP9 may play a protective role in Hcy induced VSMC lipid deposition and foam cell transformation through negative regulation of ERs. In addition, The CTRP9 promoter in the Hcy group showed hypermethylation. At the same time as Hcy intervention, overexpression of DNMT1 increases the methylation level of the CTRP9 promoter, while 5-Azc can reduce the methylation level of the CTRP9 promoter. Finally, Hcy can up-regulate the expression of DNMT1 and down-regulate the expression of CTRP9. After overexpression of DNMT1, the expression of CTRP9 is further decreased. After 5-Azc inhibition of DNMT1, the expression of DNMT1 decreases, while the expression of CTRP9 increases. It is suggested that the molecular mechanism of Hcy inhibiting the expression of CTRP9 is related to the hypermethylation of the CTRP9 promoter induced by Hcy and regulated by DNMT1. 5-Azc can inhibit the expression of DNMT1 and reverse the regulatory effect of DNMT1 on CTRP9. Overall, the results of the present study suggested that Hcy induces DNA hypermethylation in the CTRP9 promoter region by up-regulating DNMT1 expression, and negatively regulates ERs mediated VSMC lipid deposition and foam cell formation. CTRP9 may potentially be a therapeutic target in the treatment of hyperhomocysteinemia and As.

## Introduction

Foam cells are characteristic cells in atherosclerosis (As) plaques, derived from smooth muscle cells (VSMC) and macrophages^1,2^. Based on the effect of pathological factors causing atherosclerosis, VSMC migrates from the vascular tunica media to the endo subcutaneous area and transforms from contractile phenotype to secretory phenotype. After phenotype transformation, VSMC phagocytoses a large number of lipid proteins in the endovascular subcutaneous area and finally forms foam cells^3,4^. The accumulation of foam cells in the endovascular subcutaneous area is the main lesion of As, and inhibiting the formation of foam cells has become an important aspect in preventing and treating As.

Homocysteine (Hcy) is an important independent risk factor in As^5^. Our previous studies have shown that Hcy can induce the occurrence of As by inducing the proliferation, migration, and phenotypic transformation of VSMC^6^. However, in this process, whether Hcy can induce lipid deposition of VSMC and form foam cells, as well as the detailed mechanism, are the research focus of our attention. If we can elucidate the mechanism of Hcy inducing VSMC to form foam cells, it will provide an important experimental basis for the prevention and treatment of As.

The endoplasmic reticulum is a key site for posttranscriptional modification of lipid synthesis, and ER dysfunction is associated with many disorders of lipid metabolism, including As^7^. Endoplasmic reticulum stress (ERs) is caused by environmental homeostasis imbalance within the ER and can stimulate cleavage activation of ER protein SREBP-1c and SREBP-2 precursor proteins, thereby promoting synthesis of triglycerides and cholesterol, leading to lipid metabolism disorder^8,9^. There have been some reports on ERs induced by Hcy. HCY can induce inflammatory apoptosis of endothelial cells and promote the occurrence of As via activating the NF-κB signaling pathway and enhancing the ERs pathway^10^. Through the synergistic action of DNMT1 and G9a, Hcy down-regulates ERO1α expression and activates ERs and apoptosis in hepatocytes^11^. Hcy can trigger ERs in hepatocytes via down-regulating ERO1α expression, characterized by increased levels of ERs marker proteins GRP78, PERK, ATF6, and X-box binding protein-1 (XBP-1), which promoted As^12^. Hcy promotes atherosclerotic vulnerable plaque formation by activating endoplasmic reticulum stress-dependent macrophage apoptosis^13^. These results suggest that ERs-mediated lipid metabolism disorder is involved in the formation of As induced by Hcy. However, the molecular events of endoplasmic reticulum stress induced by Hcy in VSMC in the context of atherosclerosis remain unclear.

C1q/Tumor necrosis factor-related protein9 (CTRP9) is a novel endothelium-dependent and No-mediated vasodilator, which possesses similar vascular protective activity to adiponectin^14^. CTRP9 is closely related to cardiovascular diseases such as atherosclerosis, vascular calcification, pulmonary arteriosclerosis, and reverse heart remodeling^15^. CTRP9 inhibits hyperglycemia-induced endothelial senescence through AMPKα/KLF4 signaling pathway, thereby reducing As^16^. CTRP9 protects atherosclerosis by inducing autophagy promoting cholesterol effluence and reducing foam cell formation in an AMPK/mTOR signaling pathway-dependent manner^17,18^, suggesting that CTRP9 enables protection for AS. There are also some reports on the relationship between CTRP9 and ERs. CTRP9 reduces hepatic steatosis by inhibiting ERs-mediated autophagy^19^. Cardiogenic CTRP9 protects myocardial ischemia/reperfusion injury by calreticulin-dependent inhibition of ERS-related apoptosis^20^. It is suggested that CTRP9 may inhibit ERs, indicating that there is a certain relationship between CTRP9 and ERs. However, it is still unclear whether CTRP9 plays an important role in the process of Hcy inducing VSMC to form foam cells and its mechanism.

The current study aimed to uncover the association between CTRP9 and ERs and their effects on lipid deposition and foam cell formation of Hcy-induced VSMC both in vivo and in vitro. Therefore, a high Hcy apolipoprotein E deficient (ApoE^−/−^) mouse model and a Hcy-stimulated VSMC in vitro model were established. More specifically, the current study explored whether CTRP9 could serve a functional role in Hcy-induced VSMC lipid deposition and foam cell formation, possibly via regulating ERs.

## Materials and methods

### Animal treatment

Six weeks male ApoE^−/−^ mice (weight, 25–28 g. Beijing Weishang Rituo Technology Co., Ltd.) were randomly divided into the following two groups (n = 6/group): (i) ApoE^−/−^ mice fed with normal diet (ApoE^−/−^ + NC group); and (ii) ApoE^−/−^ mice fed with 1.7% methionine diet (ApoE^−/−^ + HMD group). After 15-week experimental diets, all mice were fasted overnight and anesthetized by intraperitoneal injection of pentobarbital sodium (50 mg /Kg·W), blood was taken from the eyeball, and the aorta of the mice was isolated from the heart of mice, frozen in liquid nitrogen and stored at −80 °C until further analysis. At the end of the experiment, 5% isoflurane was used for euthanasia^21,22^. All animals received humane care according to the Institutional Authority for Laboratory Animal Care of Ningxia Medical University based on the Guide for the Care and Use of Laboratory Animals published by the United States National Institutes of Health. (Ethical approval No. 2020-548).

### Detection of serum Hcy, total cholesterol, and triglyceride

Blood samples were collected from the orbital sinus and centrifuged at 3000 rpm for 10 min at 4 °C. then serum concentrations of Hcy, total cholesterol (TC), and triglyceride (TG) were measured by an automatic biochemical analyzer (Siemens, Germany) as previously described^21,22^.

### Haematoxylin and eosin (HE) and oil red O staining

HE staining and oil-red O staining were performed in frozen sections of the aortic root vessels of mice to observe the pathological changes. Details about the staining can be found in our previous study^21^, and lipid‐stained lesions were measured by digitizing morphometry and reported in mm^2^ per lesion.

### Immunofluorescence staining

Immunofluorescence staining of the frozen aortic root sections (5.0-μm thick) from ApoE^−/−^ mice was performed as previously described. Briefly, sections were fixed in cold acetone for 30 min, blocked with goat serum and stained with primary antibodies against α-smooth muscle actin (α-SMA, dilution, 1:1000, no. ab21027, Abcom), CTRP9, GRP78, ATF6a, p-PERK, p-IRE1α, SREBP1c, SREBP2 (all from Affinity) at 4 °C overnight. Images were captured under laser scanning confocal microscopy (Olympus, Japan) and colocalization analysis was performed using the Coloc 2 plugin in ImageJ^23^.

### Cell transfection

The sequences of CTRP9 overexpression plasmid, GFP, Si-NC, and Si-CTRP9 were obtained from Genepharma (Shanghai, China) and they were transiently transfected into the cells using Lipofectamine 2000 (Life Technologies, Gaithersburg, MD, USA) following the manufacturer's instruction^24^. The method was as follows: 5 × 10^5^ cells per well were inoculated into the 96-well culture plate the day before transfection, and the cell fusion degree reached 90–95%. The transfer solution 100 μl containing plasmid transfection reagents was added to the culture plate and cultured at 37 °C in an incubator with 5% CO_2_. After 6 h, the medium was replaced and the culture was continued for 24 h. The transfection efficiency was detected by qRT-PCR and western blot, then the cells were collected for downstream analysis.

### Construction of recombinant DNMT1 adenoviruses

The human DNMT1 gene was inserted into the replication-defective adenoviral shuttle vector pHBAd-CMV-IRES-GFP. The DNMT1 expressing construct was co-transfected with adenoviral backbone plasmid pBHGlox (Delta) E1, 3Cre into virus packaging cell line 293. The virus was collected and used for infection of VSMC. The adenovirus encoding the green fluorescent protein (Ad-GFP) was used as a control. Infected cells that were more than 80% positive for GFP were used for further experiments. Western blotting was carried out to examine ectopic gene expression using antibodies against DNMT1.

### Cell culture and treatment

Human VSMC were purchased from the BeNa Culture Collection (BNCC; Suzhou Bena Chuanglian Biotechnology Co. Ltd.) and cultured in DMEM (Gibco, Grand Island, NY, USA) supplemented with 7% fetal bovine serum (FBS; Gibco; Thermo Fisher Scientific, Inc.) and 1% penicillin/streptomycin solution (Beijing Solarbio Science & Technology Co., Ltd.) at 37 °C in an incubator with 5% CO_2_^21^. VSMC were divided into the normal Control (Hcy-free), Hcy (100 μmol/L), Hcy + Si-NC, Hcy + Si-CTRP9, Hcy + Si-CTRP9 + 4-PBA (10 mmol/L, ERs inhibitor, MCE, HY-A0281), Hcy + GFP, Hcy + CTRP9, Hcy + CTRP9 + TM (0.5 μg/mL, ERs agonist, MCE, HY-A0098) groups. All cells used were between passages 3 and 7. Prior to each experiment, VSMC was induced with 100 μmol/L Hcy for 48 h. HE staining and oil red O staining were used to observe the effect of 100 umol/L Hcy stimulation on lipid deposition in VSMC. In addition, the levels of TC and TG in VSMC were measured using an automatic biochemical analyzer.

### Reverse transcription-quantitative PCR (RT-qPCR)

Total RNA was extracted from VSMC using the RNA simple Total RNA kit (Tiangen Biotech Co., Ltd.), according to the manufacturer's instructions. Subsequently, 1 µg total RNA was reverse transcribed into cDNA using cDNA Reverse Transcription kits (Thermo Fisher. Scientific, Inc.) The mRNA expression levels of CTRP9, GRP78, ATF6a, PERK, IRE1α, SREBP1c and SREBP2 were detected by qPCR using a SYBR green PCR Kit (DBI Bioscience) on the LightCycler System (Roche Applied Science). The primer sequences used are listed in Table 1. The thermocycler conditions were as follows: 37 °C for 30 s; 95 °C for 15 min; followed by 40 cycles of 95 °C for 10 s; 55 °C for 30 s; and 72 °C for 30 s. The obtained Ct values were analyzed based on the amplification curves and the relative expression levels of the target genes were calculated using the 2^−ΔΔCq^ method^25^. PCR reactions were performed in triplicate and normalized using β-actin as a reference gene.

**Table 1:** Sequence information of primers used in RT-qPCR.

Gene	Primers sequence	Tm (°C)	Size (bp)
*CTRP9*	F: 5′-**GGATGGGACGAGTGGAGAGAA**-3′	62	113
	R: 5′-**CCTTGGGGCCATGTTTTCCT**-3′		
*GRP78*	F: 5′-**CATCACGCCGTCCTATGTCG**-3′	59	104
	R: 5′-**CGTCAAAGACCGTGTTCTCG**-3′		
*ATF6a*	F: 5′-**TCCTCGGTCAGTGGACTCTTA**-3′	56	235
	R: 5′-**CTTGGGCTGAATTGAAGGTTTTG**-3′		
*PERK*	F: 5′-**GACCTGAAGCCCTCCAATCT**-3′	59	170
	R: 5′-**CACATACTCGGTCAGAAAGCC**-3′		
*IRE1a*	F: 5′-**CACAGTGACGCTTCCTGAAAC**-3′	56	169
	R: 5′-**GCCATCATTAGGATCTGGGAGA**-3′		
*SREBP1c*	F: 5′-**CGGAACCATCTTGGCAACAGT**-3′	60	141
	R: 5′-**CGCTTCTCAATGGCGTTGT**-3′		
*SREBP2*	F: 5′-**AACGGTCATTCACCCAGGTC**-3′	55	133
	R: 5′-**GGCTGAAGAATAGGAGTTGCC**-3′		
*β-actin*	F: 5′-**CATGTACGTTGCTATCCAGGC**-3′	56	250
	R: 5′-**CTCCTTAATGTCACGCACGAT**-3′		

*F* forward, *R* reverse, *CTRP9* C1q/tumor necrosis factor-related protein9, *GRP78* glucose regulated protein 78 kD, *ATF6a* activating transcription factor 6a, *PERK* protein kinase R-like ER kinase, *IRE1* αinositol-requiring enzyme-1α, *SREBP1c* sterol regulatory element binding proteins-1c, *SREBP2* sterol regulatory element binding proteins-2.

### Western blot analysis

To detect the changes in the protein levels in VSMC, western blot analysis was carried out using specific antibodies^6^. Total proteins were isolated from cells using a Whole protein extraction kit (Nanjing KeyGen Biotech Co., Ltd.), while protein concentration was determined using the SimpliNano™ Biochrom Spectrophotometer (Biochrom, Ltd.). The protein samples (20 μl/lane) were separated by SDS-PAGE and were then transferred onto a PVDF membrane (MilliporeSigma). Following blocking with 5% non-fat milk in PBS with Tween-20, the membrane was incubated at 4 °C overnight with the following antibodies: Anti-CTRP9 (dilution, 1:1000, no. DF9407), anti-GRP78 (dilution, 1:1000, no. AF5366), anti-AFT6a (dilution, 1:1000, no. DF6009), anti-PERK (dilution, 1:800, no. AF5304), anti-phospho(p)**-**PERK (dilution, 1:1000, no. DF7576), anti-IRE1a (dilution, 1:1000, no. DF7709), anti-phospho(p)**-**IRE1a (dilution, 1:1000, no. AF7150), anti-SREBP1c (dilution, 1:800, no. AF6283), anti-SREBP2 (dilution, 1:1000, no. DF7601.), anti-DNMT1 (dilution, 1:1000, no. DF9407) and anti-β-actin (dilution, 1:1000, no. AF7018, all from affinity). Following washing, the membranes were incubated with the corresponding horseradish peroxidase-conjugated IgG (anti-rabbit, no. ZB2301 or anti-mouse, no. ZB2305, dilution, 1:5000. ZSGB-BIO) for 4 h. Finally, the protein bands were visualized using chemiluminescence (ECL; Nanjing KeyGen Biotech Co., Ltd.).

### Bisulfite sequencing PCR (BSP)

VSMC were divided into the normal Control, Hcy, Hcy + 5-Azc (5 μmol/L, MCE, HY-10586), Hcy + Ad-GFP, and Hcy + Ad-DNMT1 groups. The methylation status of CpG sites within the CTRP9 promoter was examined by bisulfite sequencing using genomic DNA extracted from VSMC cells by a QIAamp DNA Mini Kits (Qiagen, 51304, Mississauga, ON, CA). Bisulfite conversion was performed on 2 μg of DNA using an EpiTect Bisulfite (Qiagen, 59104, Mississauga, ON, CA). The bisulfite-treated CTRP9 promoter containing 30 CpG sites was amplified with the primers 5′-GTGGTTGGGTTGGAGTGTGTAGA-3′ (sense) and 5′-CAAAAAACCCCTAAATCCCAACT-3′ (antisense). PCRs were performed using GoTaq HotStart Polymerase (Promega) as follows: 98 °C for 2 min, 30 cycles of 98 °C for 10 s, 55 °C for 10 s, and 72 °C for 15 s, followed by a final extension at 72 °C for 5 min. The amplified PCR products were purified and subcloned into a pGEM T-Easy vector (Promega, Madison, WI). Plasmid-transformed DH5a bacteria were cultured overnight, and the plasmid DNA was isolated (Axygen, California, USA). At least ten separate clones were selected for sequence and their methylation status was analyzed using web-based analysis software QUMA (http://quma.cdb.riken. jp/)^26^. The percentage of methylated CpG dinucleotides was calculated to evaluate the methylation level of CTRP9.

### Statistical analyses

All statistical analyses were performed using GraphPad Prism 5.0 software. Data are expressed as the mean ± standard deviation (SD). SD of at least three independent experiments. The differences in single parameters between the two groups were compared using paired Student's t-test, while those among multiple groups using Kruskal–Wallis one-way ANOVA, followed by Dunn's test. A P-value ≤ 0.05 was considered to indicate a statistically significant difference.

### Ethical approval

The study was approved by the Ethics Committee of Ningxia Medical University (NO. 2020-548) and conducted by the Guide for the Care and Use of Laboratory Animals, and the reporting follows the recommendations in the ARRIVE guidelines.

## Results

### Hcy induced the transformation of VSMC into foam cells in the aortic root of mice

**Figure 1:**
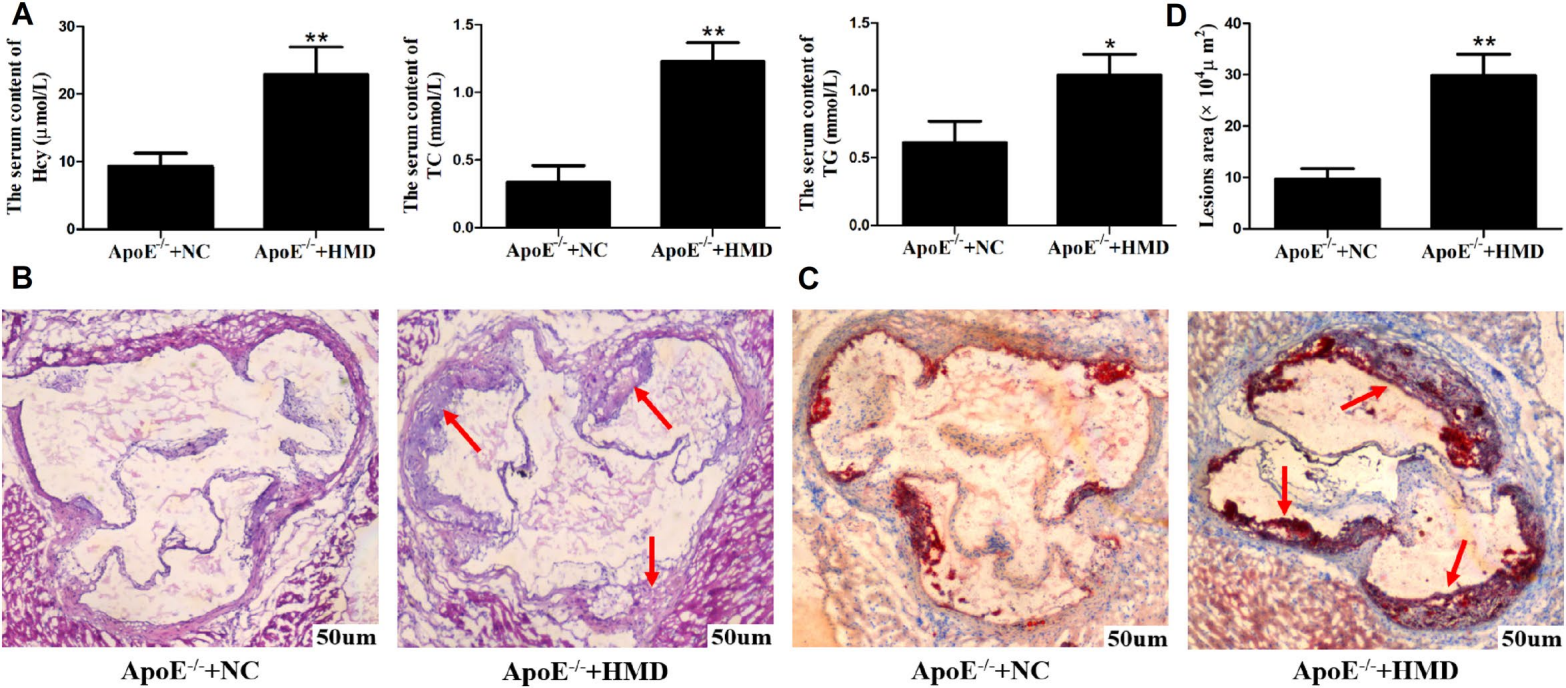
Pathological changes of aortic vessels in mice and changes of serum Hcy, TC, and TG contents. (**A**) Changes of serum Hcy, TC, and TG contents in mice. (**B**) HE staining of frozen sections of blood vessels in the aortic root of mice. The "red arrow" indicates that the smooth muscle layer of the vascular medium membrane contains a large number of lipid vacuoles, which are thickened and disordered, protruding significantly into the blood vessels to form lipid plaques. (**C**) Oil red O staining of frozen sections of the aorta root vessels of mice. In ApoE^−/−^ + HMD group, there were obvious As plaques in the vascular wall. In the atherosclerotic plaques, the lipids were bright red, the nuclei were blue, the interstitium was colorless and the structure was clear. (**D**) Oil red O staining area comparison. The experiment was performed in triplicate, and the representative images are shown. *P < 0.05, **P < 0.01 compared with the ApoE^−/−^ + NC group.

To determine whether ApoE^−/−^ mice high Hcy As animal model was successfully replicated, the contents of serum Hcy, TC, and TG were quantitatively analyzed by the automatic biochemical analyzer. The results showed that the contents of serum Hcy, TC, and TG of ApoE^−/−^ mice fed 1.7% high methionine diet were significantly higher than those of the normal diet group (Fig. 1A), suggesting that high Hcy could exacerbate the disorder of lipid metabolism. The serum Hcy content was more than 20 μmol/L, indicating that the animal model of hyperhomocysteinemia was successfully replicated.

To identify the changes of VSMC in ApoE^−/−^ mice HHcy animal model following a high methionine diet, frozen sections of blood vessels in the aortic root of mice were stained with HE staining (Fig. 1B) and oil red O staining (Fig. 1C, D) to observe the pathological changes. In ApoE^−/−^ mice with normal diet (ApoE^−/−^ + NC) group, As plaques were found in the aorta root vessels, the inner and middle membranes were slightly thickened and the inner elastic membranes were clear. In ApoE^−/−^ mice fed a high methionine diet (ApoE^−/−^ + HMD) group, a large number of As plaques were formed in the aorta root vessels, with a large area of plaques, obvious thickening and disordered arrangement of the smooth muscle layer of the medium membrane, and migrated through the inner elastic membrane to the subintima, and a large number of vacuoles were observed, as shown in the red arrow. It is suggested that high methionine diet leads to the formation of a large number of fat vacuoles in VSMC and the migration of VSMC through the inner elastic membrane to the subintimal membrane. Oil-red O staining showed a small number of scattered red lipid droplets in the vascular wall of the ApoE^−/−^ + NC group. In the ApoE^−/−^ + HMD group, obvious As plaques could be seen in the vascular wall, with large amounts of bright red lipids, blue nuclei, colorless interstitium, and clear structure, as shown by the red arrow. Image J software was used to calculate the oil red O staining area of the two groups, and it could be seen that the oil red O staining area of the ApoE^−/−^ + HMD group increased significantly. These results suggested that Hcy stimulated the formation of lipid plaques in aortic vessels, and induced large amounts of lipid deposition in VSMC, which were transformed into foam cells.

### Expression of CTRP9, GRP78, ATF6a, p-PERK, p-IRE1α, SREBP1c, and SREBP2 in the aortic root of mice

**Figure 2:**
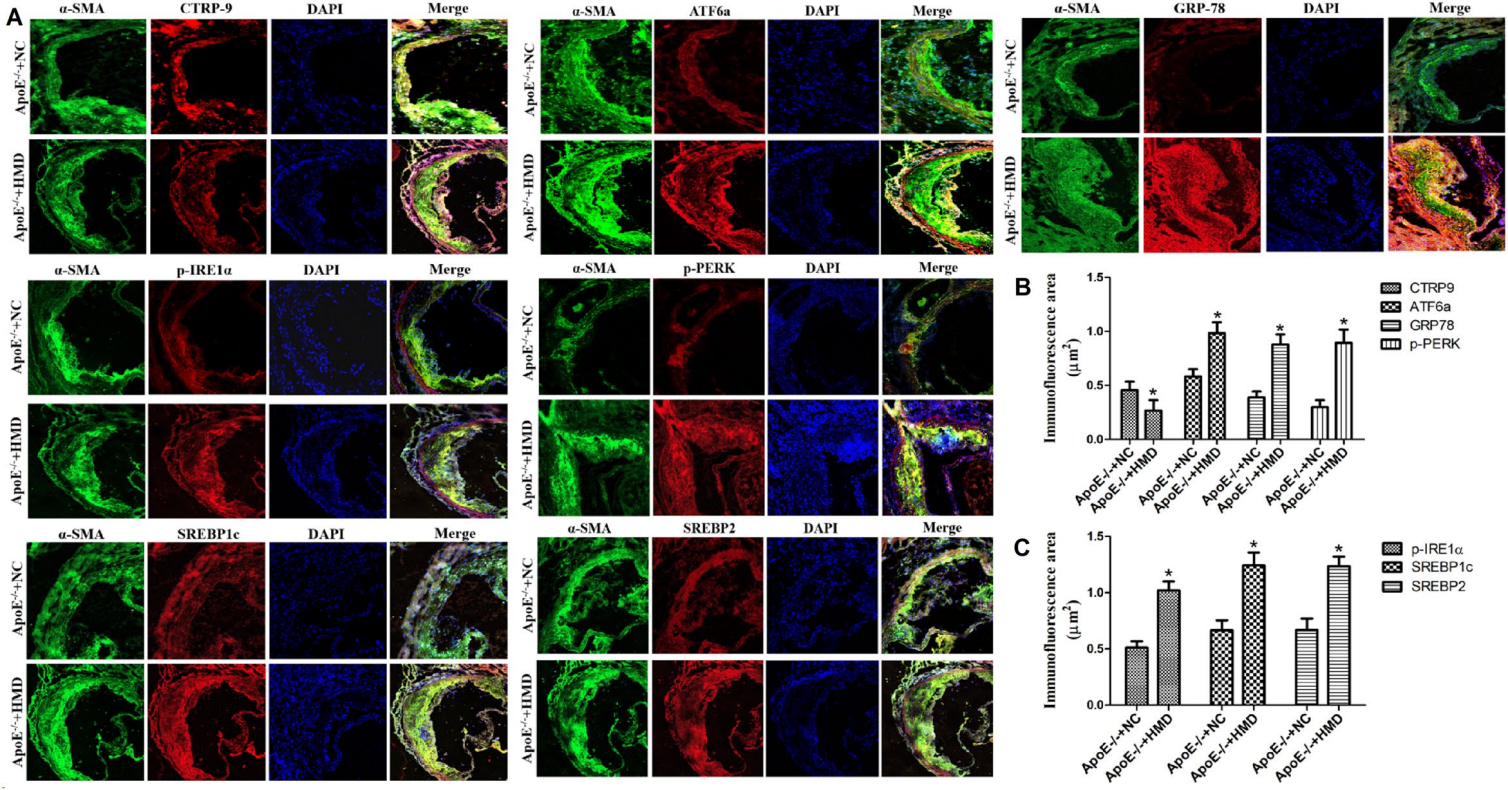
Expression of CTRP9, GRP78, ATF6a, p-PERK, p-IRE1α, SREBP1c and SREBP2 in the aortic root of mice. (**A**) Immunofluorescence staining of CTRP9, GRP78, ATF6a, p-IRE1α, p-IRE1α, SREBP1c, and SREBP2 with α-SMA in the VSMC of the aortic root of mice. The photos are representative images from three separate experiments. The blue fluorescence is the nucleus of vascular smooth muscle cells (DAPI), and the green and red fluorescence is the target protein (×40). (**B**) Immunofluorescence staining area analysis of CTRP9, GRP78, ATF6a, and p-IRE1α. (**C**) Immunofluorescence staining area analysis of p-IRE1α, SREBP1c, and SREBP2. The experiment was performed in triplicate, and the representative images are shown. *P < 0.05, **P < 0.01 compared with the ApoE^−/−^ + NC group.

To investigate whether the mechanism of Hcy-induced VSMC foaming change is related to CTRP9 and ERs, we performed immunofluorescence staining on ApoE^−/−^ mice aortic root frozen sections and observed the expression of the CTRP9, GRP78, ATF6a, p-IRE1α, p-PERK, SREBP1c and SREBP2 in aortic VSMC (Fig. 2). The blue fluorescence is the nucleus of VSMC (DAPI), the green fluorescence is the protein expression of α-SMA, and the red fluorescence is the protein expression of CTRP9, GRP78, ATF6a, p-PERK, p-IRE1α, SREBP1c and SREBP2. After Image J software analysis, the immunofluorescence staining area of CTRP9 in aorta VSMC of the ApoE^−/−^ + HMD group was significantly reduced, and the immunofluorescence staining area of GRP78, ATF6a, p-IRE1α, p-IRE1α, SREBP1c, and SREBP2 increased significantly. These results indicated that the expression of CTRP9 protein in mouse aorta VSMC was decreased, while the expression of ERs marker proteins GRP78, ATF6a, p-IRE1α, p-IRE1α, and intracellular lipid homeostasis regulatory factors SREBP1c and SREBP2 were increased. These results indicate that high Hcy stimulates the low expression of CTRP9 in mouse aorta VSMC, while ERs and lipid metabolism disorders occur.

### CTRP9 negatively regulates ERs to inhibit the Hcy-induced transformation of VSMC into foam cells

**Figure 3:**
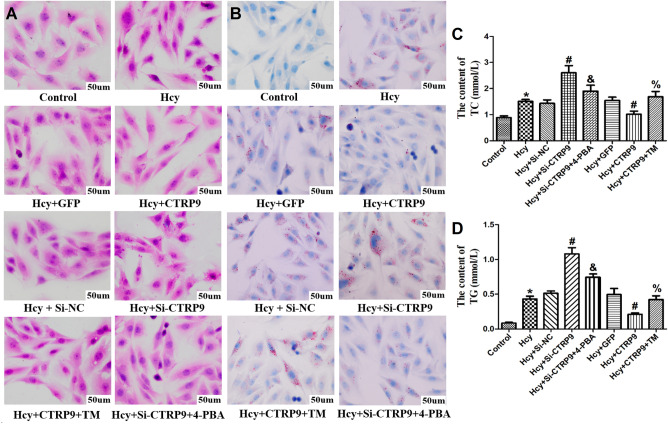
Changes of lipid contents in VSMC in each group. (**A**) HE staining of VSMC in each group. (**B**) Oil red O staining of VSMC in each group. The nucleus is blue; The fat drops are red. (**C**) Changes of TC contents in VSMC in each group. (**D**) Changes of TG content in VSMC of each group. The experiment was performed in triplicate, and the representative images are shown. *P < 0.05 compared with the control group. ^#^*P* < 0.05 compared with the Hcy group. ^%^*P* < 0.05 compared with the Hcy + CTRP9 group. ^&^*P* < 0.05 compared with the Hcy + Si-CTRP9 group.

To explore whether the lipid deposition and foam cell formation of VSMC induced by Hcy are related to the CTRP9 and ERs, HE staining (Fig. 3A) and oil red O staining (Fig. 3B) were used to observe the effects of 100 umol/L Hcy stimulation on lipid deposition in VSMC. The results showed that a large number of vacuoles and red lipid droplets could be seen in VSMC of the Hcy group, while the contents of TC (Fig. 3C) and TG (Fig. 3D) were significantly increased. These results suggested that Hcy stimulation promoted lipid deposition in VSMC. After transfection of CTRP9, VSMC hollow vacuoles and red lipid droplets were significantly reduced, and the contents of TC and TG were significantly decreased. After Si-CTRP9, VSMC hollow bubble and red lipid droplets increased significantly, while TC and TG contents increased significantly, suggesting that the down-regulation of CTRP9 expression is related to Hcy-induced lipid deposition in VSMC, and the increased expression of CTRP9 has a protective effect on Hcy induced lipid deposition in VSMC.

After overexpression of CTRP9, ERs agonist TM was given to stimulate VSMC cells, VSMC hollow vacuoles and red lipid droplets increased significantly, and the contents of TC and TG in VSMC increased significantly. When the ERs inhibitor 4-PBA was given at the same time as Si-CTRP9 to stimulate VSMC cells, VSMC hollow vesicles and red lipid droplets were significantly reduced, and the contents of TC and TG in VSMC were significantly decreased. It is suggested that ERs can promote Hcy induced VSMC lipid deposition, inhibition of ERs can reduce Hcy induced VSMC lipid deposition, and CTRP9 may play a protective role in Hcy induced VSMC lipid deposition and foam cell transformation by negatively regulating ERs.

### Effect of CTRP9 on the expression of ERs marker protein and lipid homeostasis regulatory factor in VSMC

**Figure 4:**
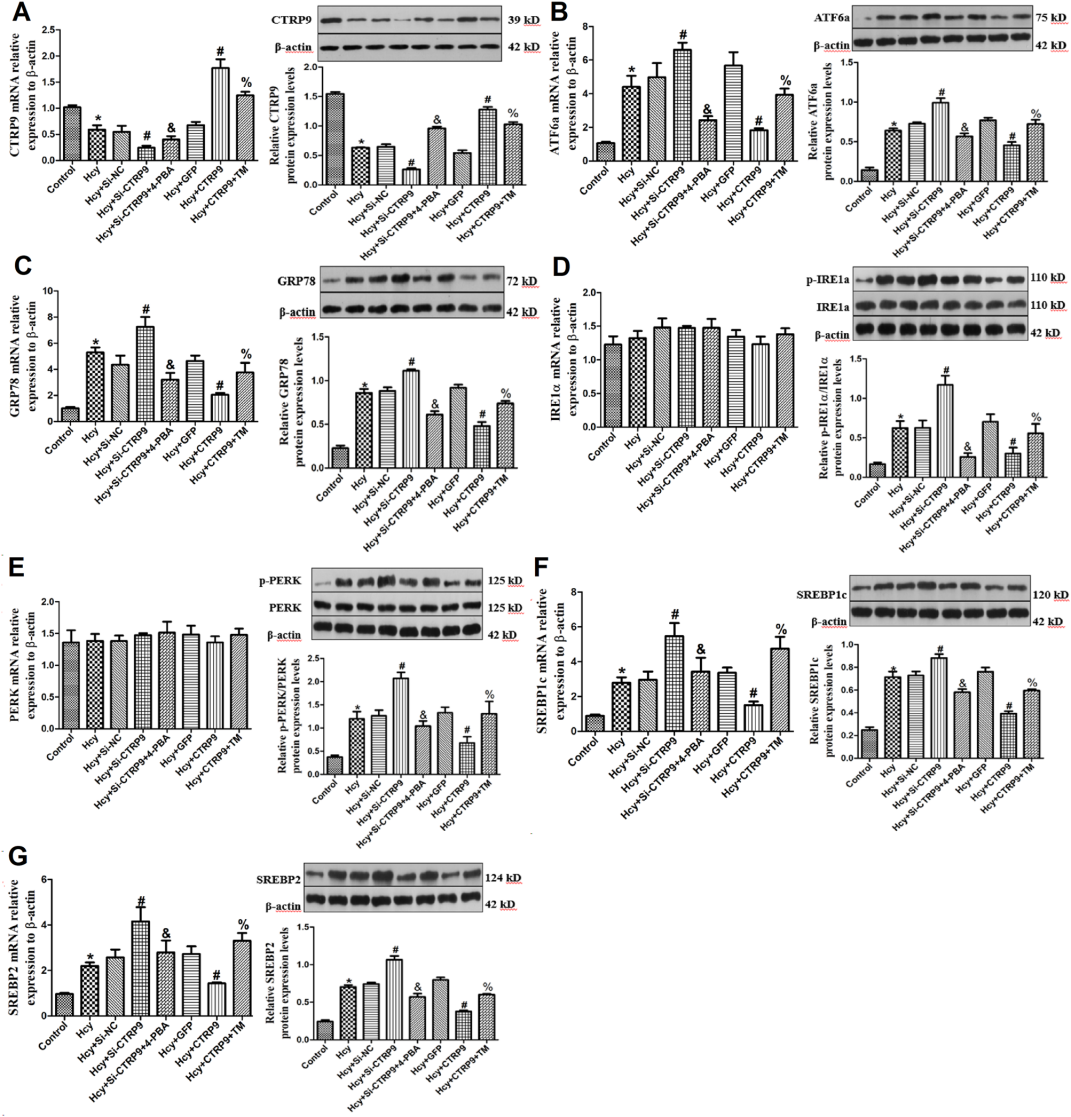
Effects of CTRP9 on the expression of ERs marker proteins and lipid homeostasis regulatory factors in VSMC. (**A**) CTRP9 mRNA and protein expression levels. (**B**) ATF6a mRNA and protein expression levels. (**C**) mRNA and protein expression levels of CRP78. (**D**) Expression levels of IRE1a mRNA and p-IRE1a protein. (**E**) Expression levels of PERK mRNA and p-PERK protein. (**F**) SREBP1c mRNA and protein expression levels. (**G**) SREBP2 mRNA and protein expression levels. The experiment was performed in triplicate, and the representative images are shown. *P < 0.05 compared with the control group. ^#^*P* < 0.05 compared with the Hcy group. ^%^*P* < 0.05 compared with the Hcy + CTRP9 group. ^&^*P* < 0.05 compared with the Hcy + Si-CTRP9 group.

To explore the specific mechanism of CTRP9 negatively regulating ERs, the expression of ERs marker protein and lipid homeostasis regulatory factor in VSMC were detected by qRT-PCR and Western blot. Compared with the Control group, the mRNA and protein expression of CTRP9 in VSMC in the Hcy group were significantly decreased (Fig. 4A). The mRNA and protein expressions of GRP78, ATF6a, SREBP1c, and SREBP2 were significantly increased, and the mRNA and protein expressions of PERK and IRE1α were not significantly changed, but the protein expressions of p-PERK and p-IRE1α were significantly increased (Fig. 4B–G). It is suggested that Hcy can down-regulate the expression of CTRP9, activate ERs, and cause lipid deposition in VSMC. After Si-CTRP9, the expressions of GRP78, ATF6a, p-PERK, p-IRE1α, SREBP1c and SREBP2 were significantly increased. After CTRP9 is overexpressed, the opposite result is obtained (Fig. 4B–G). It is suggested that CTRP9 plays a regulatory role in ERs and lipid metabolism of VSMC, and the down-regulation of CTRP9 activates ERs and the imbalance of lipid homeostasis of VSMC.

After Si-CTRP9, ERs inhibitor 4-PBA was given to stimulate VSMC cells, and the mRNA and protein expression of CTRP9 in VSMC were significantly increased (Fig. 4A). The expressions of GRP78, ATF6a, p-PERK, p-IRE1α, SREBP1c and SREBP2 were significantly decreased (Fig. 4B–G). While after the overexpression of CTRP9, ERs agonist TM was given to stimulate VSMC cells, the expression of CTRP9 mRNA and protein in VSMC was significantly decreased (Fig. 4A), while GRP78, ATF6a, P-perk, p-IRE1α, SREBP1c and SREBP2 were significantly increased (Fig. 4B–G). These results indicate that Hcy can inhibit the expression of CTRP9 in VSMC and induce ERs and lipid deposition. At the same time CTRP9 can negatively regulate ERs, reduce lipid production in VSMC, and maintain lipid metabolic homeostasis in VSMC.

### Hcy induces hypermethylation of CTRP9 promoter via DNMT1

Previous research findings that Hcy can increase the mRNA and protein expression of DNMT1 in VSMC and promote the proliferation of Hcy-induced VSMC^21,23^. These results suggest that Hcy can induce DNA methylation regulation in VSMC. However, it is not clear whether the inhibition of CTRP9 expression by Hcy is also achieved through epigenetic regulation.

**Figure 5:**
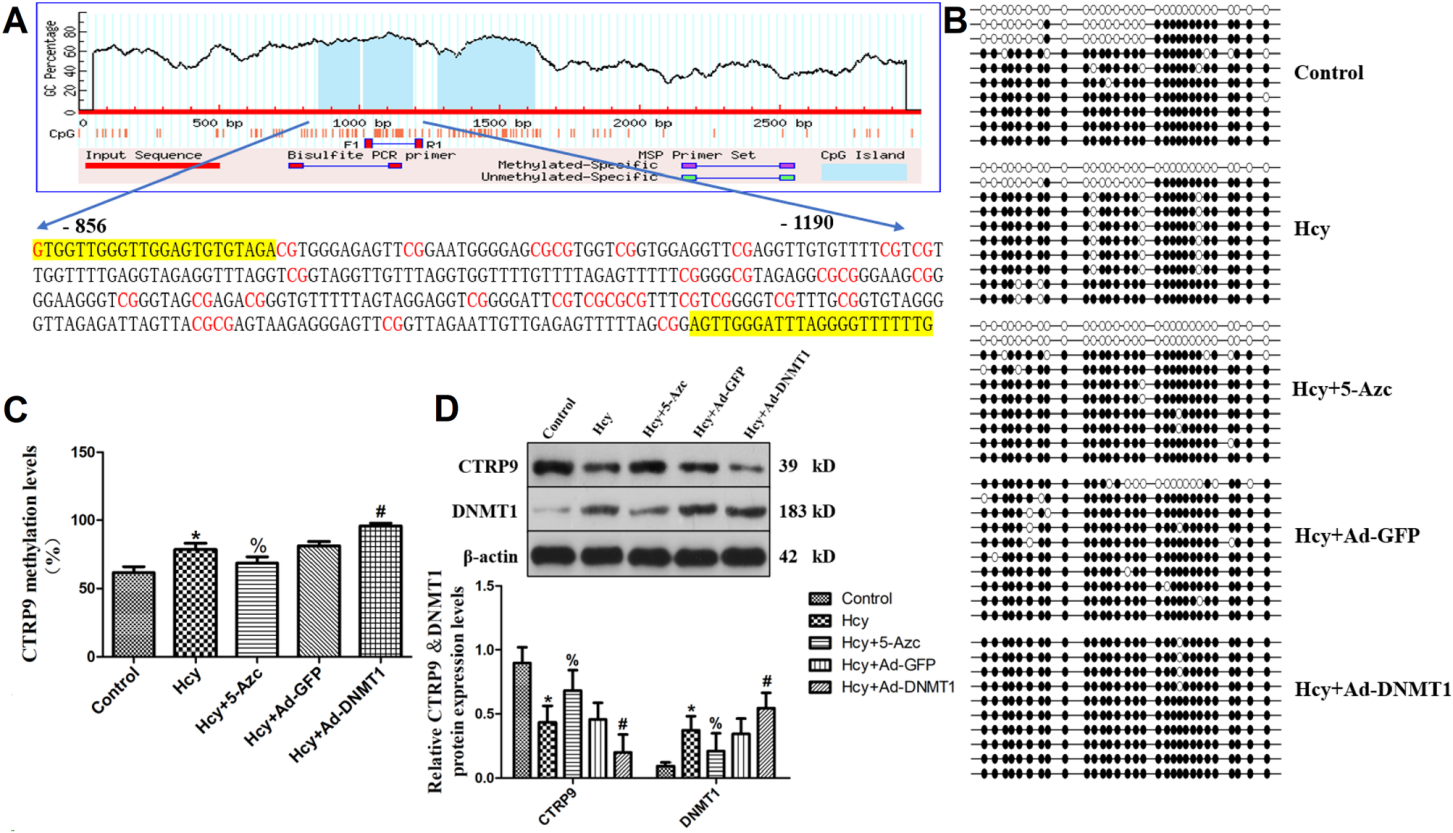
Hypermethylation of CTRP9 promoter induced by Hcy via DNMT1. (**A**) Prediction of CpG islands in CTRP9 promoter region and design of specific primers for both ends of CpG islands. (**B**) Dot plots of VSMC methylation sequencing results in each group, with black solid dots representing methylation and white hollow dots representing non-methylation. (**C**) Comparison of methylation levels of CTRP9 promoter in VSMC of each group. (**D**) Expression changes of CTRP9 and DNMT1 in VSMC of each group. The experiment was performed in triplicate, and the representative images are shown. *P < 0.05 compared with the control group. ^#^*P* < 0.05 compared with the Hcy group. ^%^*P* < 0.05 compared with the Hcy + DNMT1 group.

To clarify the specific mechanism of Hcy inhibiting CTRP9 expression, we studied the methylation modification of the CTRP9 promoter region. The NCBI database was used to search the CTRP9 promoter region, and CpG Island was used to predict the methylation site. The results showed that there were 2 CpG islands in the CTRP9 promoter region (−856 bp to −1190 bp, −1283 bp to −1625 bp), among which the region from −856 to −1190 bp was the CpG island with the highest density of CG sites, which contained a total of 30 CpG dinucleotides (Fig. 5A), suggested that CTRP9 may be regulated by methylation. To further investigate whether the inhibition of CTRP9 expression by Hcy is regulated by methylation and the effect of DNMT1 after Hcy stimulates VSMC, recombinant adenovirus expressing DNMT1 is transfected into VSMC or DNMT1 inhibitor 5-Azc is given to interfere with VSMC. Assess CTRP9 promoter methylation levels by bisulfite sequencing PCR. The results showed (Fig. 5B,C ) that the CTRP9 promoter in the Hcy group showed hypermethylation. At the same time as Hcy intervention, overexpression of DNMT1 increases the methylation level of the CTRP9 promoter, while administration of DNMT1 inhibitor 5-Azc can reduce the methylation level of the CTRP9 promoter. These results suggest that DNA methylation plays an important role in the lipid deposition of VSMC induced by Hcy. The molecular mechanism of Hcy inhibiting CTRP9 expression is related to the hypermethylation of the CTRP9 promoter region induced by Hcy, and DNMT1 plays an important role in this process. To confirm the relationship between DNMT1 and CTRP9 hypermethylation, the expressions of DNMT1 and CTRP9 were detected by western blot. The results show (Fig. 5D) that Hcy can up-regulate the expression of DNMT1 and down-regulate the expression of CTRP9. After overexpression of DNMT1, the expression of CTRP9 is further decreased, suggesting that the inhibitory effect of Hcy on CTRP9 is regulated by DNMT1. After the 5-Azc intervention, the expression of DNMT1 decreased, but the expression of CTRP9 increased. It is suggested that 5-Azc can inhibit the expression of DNMT1 and reverse the regulatory effect of DNMT1 on CTRP9.

## Discussion

In this study, it is found for the first time that CTRP9 can reduce the lipid deposition of Hcy-induced VSMC and inhibit the transformation of VSMC into foam cells by negatively regulating ERs, thus playing a protective role in the pathogenesis of As caused by Hcy, which has not been reported in previous studies. At the same time, the main mechanism of the down-regulation of CTRP9 expression in VSMC induced by Hcy is related to the hypermethylation of the CTRP9 promoter region, and DNMT1 plays an important role in this process. CTRP9 may be a potential therapeutic target for the prevention and treatment of hyperhomocysteinemia and As.

Atherosclerosis (As) is a chronic compensatory arterial inflammatory response associated with abnormal lipid metabolism and changes in the composition of blood vessel walls, which is the main cause of cardiovascular disease (CVD)^27^. Vascular smooth muscle cell (VSMC) is the main cellular component in As plaques and one of the main sources of foam cells. Literature has shown that in plaque formation of As, 70% of plaque components are composed of VSMC and their derivatives^28^, and 40% of foam cells, which constitute an important part of lesions, are from VSMC, known as smooth muscle-derived foam cells^29,30^. However, the mechanism of lipid deposition in VSMC remains unclear.

In the present study, we observed significant increases in serum Hcy, TC, and TG contents of ApoE^−/−^ mice fed a high methionine diet. Moreover, lipid plaques were formed in the aorta of mice. In vitro, Hcy induced large lipid deposition in VSMC, suggesting the transformation of VSMC into foam cells. As an intermediate product of methionine metabolism, Hcy does not directly participate in lipid metabolism. Why can Hcy significantly disturb the balance of lipid transport in the blood vessel wall, cause the disorder of lipid metabolism, promote the foaming changes of VSMC and eventually lead to As? This is the core problem in unraveling the pathogenesis of Hcy-induced As.

We used ApoE^−/−^ mice to construct an animal model of hyperhomocysteinemia As, and stimulated VSMC with 100 μmol/L Hcy in vitro. It was found that CTRP9 protein expression in VSMC was decreased, and ERs marker proteins GRP78, ATF6a, p-PERK p-IRE1α, and intracellular lipid homeostasis regulators SREBP1c and SREBP2 were elevated. These results indicated that Hcy inhibited the expression of CTRP9 in VSMC, and activated the ERs pathway and lipid metabolism disorder. Meanwhile, overexpression of CTRP9 can reduce Hcy-induced lipid deposition in VSMC, while inhibition of CTRP9 can aggravate Hcy-induced lipid deposition in VSMC. Stimulation of VSMC cells by ERs agonist TM and overexpression of CTRP9 can aggravate lipid deposition in VSMC cells, whereas stimulation of VSMC cells by ERs inhibitor 4-PBA while administration of Si-CTRP9 results in the opposite effect. While CTRP9 is overexpressed in VSMC, ERs agonist TM is given to stimulate VSMC cells, which can aggravate lipid deposition in VSMC. The opposite result was obtained when the ERs inhibitor 4-PBA was given to stimulate VSMC cells while Si-CTRP9 was administered in VSMC. It is suggested that ERs can promote Hcy-induced lipid deposition in VSMC, and inhibition of ERs can reduce Hcy-induced lipid deposition in VSMC, and CTRP9 may play a protective role in Hcy-induced lipid deposition and foam cell transformation in VSMC by negatively regulating ERs.

To further explore the specific mechanism of CTRP9 negatively regulating ERs, the expressions of ERs marker protein and lipid homeostasis regulatory factor in VSMC were detected by qRT-PCR and Western blot. The results indicated that Hcy could inhibit the expression of CTRP9 in VSMC and induce ERs and lipid deposition. CTRP9 can negatively regulate ERs, reduce lipid production in VSMC, and maintain lipid metabolic homeostasis in VSMC. However, the mechanism of CTRP9 down-regulation induced by Hcy remains unclear.

DNA methylation, a form of epigenetic regulation, refers to the binding of a methyl group to a cytosine 5' carbon covalent bond of a genomic CpG dinucleotide under the action of DNA methyltransferase (DNMTs)^31^. In the VSMC proliferative region of As plaques, the overall cytosine methylation level of the VSMC genome was reduced by 9%, suggesting that genomic hypomethylation may also play a role in smooth muscle cell proliferation^32^. However, the role of NA methylation in Hcy-induced lipid deposition of VSMC and its transformation into foam cells remains unclear.

In our experiment, it was found that the CTRP9 promoter region of the Hcy group showed hypermethylation. At the same time as Hcy intervention, overexpression of DNMT1 can increase the methylation level of the CTRP9 promoter, while administration of DNMT1 inhibitor 5-Azc can reduce the methylation level of the CTRP9 promoter. Furthermore, Hcy can up-regulate the expression of DNMT1 and down-regulate the expression of CTRP9. After overexpression of DNMT1, the expression of CTRP9 is further decreased, suggesting that the inhibitory effect of Hcy on CTRP9 is regulated by DNMT1. After 5-Azc intervention, the expression of DNMT1 decreased, but the expression of CTRP9 increased. It is suggested that 5-Azc can inhibit the expression of DNMT1 and reverse the regulatory effect of DNMT1 on CTRP9. These results confirm that DNA methylation plays an important role in the lipid deposition of VSMC induced by hyperhomocysteinemia. The molecular mechanism of Hcy inhibiting CTRP9 expression is related to the hypermethylation of the CTRP9 promoter region induced by Hcy, and DNMT1 plays an important role in this process.

To aid in the prevention and treatment of hyperhomocysteinemia (HHcy) and As, this study has uncovered the molecular mechanism by which Hcy induces lipid deposition and transformation into foam cells in VSMC. Specifically, CTRP9 is an important target gene in the transformation of endoplasmic reticulum stress regulation VSMC into foam cells in the process of Hcy-induced As. Hcy induces DNA hypermethylation in the CTRP9 promoter region through up-regulation of DNMT1 expression and negatively regulates ERs mediated lipid deposition and transformation into foam cells in VSMC. CTRP9 may be a potential therapeutic target for the prevention and treatment of hyperhomocysteinemia and As, providing a theoretical basis for the study of the pathogenesis of Hcy-induced As and the prevention and treatment of hyperhomocysteinemia and As (Supplementary Information).

### Supplementary Information

Supplementary Figures.

## Data Availability

The analyzed data and material sets generated during the present study are included in this published article.
