# Supplementary material for: Hypermethylation of the CTRP9 promoter region promotes Hcy induced VSMC lipid deposition and foam cell formation via negatively regulating ER stress
Source: Sci Rep. 2023 Nov 9;13:19438. doi: 10.1038/s41598-023-46981-5 (PMC10636064; doi:10.1038/s41598-023-46981-5)
Supplement: Supplementary file 1 — Supplementary Figures. [file 41598_2023_46981_MOESM1_ESM.pdf]

Fig. 4. Effects of CTRP9 on the expression of ERs marker proteins and lipid homeostasis regulatory factors in VSMC

(A) CTRP9 mRNA and protein expression levels.

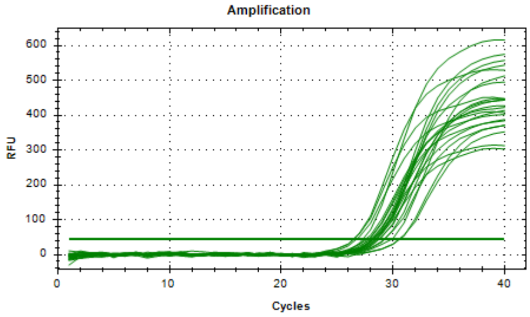

CTRP9 amplification curve

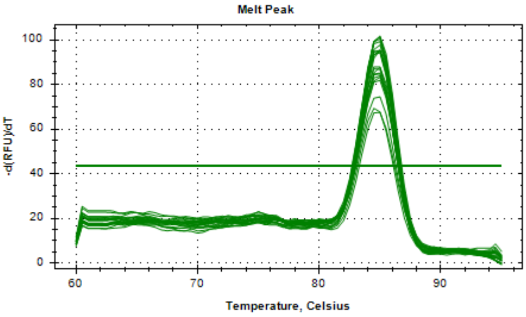

CTRP9 solution curve

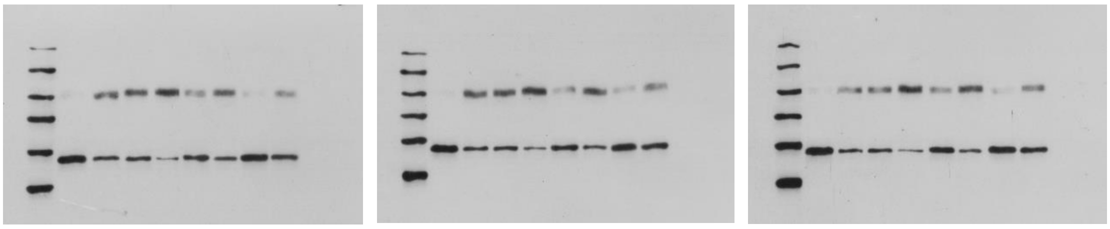

The Original blots/gels in turn are Control (Hcy-free), Hcy (100 μmol/L), Hcy+Si-NC, Hcy+Si-CTRP9, Hcy+Si-CTRP9+4-PBA (10 mmol/L, ERs inhibitor, MCE, HY-A0281), Hcy+GFP, Hcy+CTRP9, Hcy+CTRP9+TM (0.5 μg/mL, ERs agonist, MCE, HY-A0098) groups.

(B) ATF6a mRNA and protein expression levels.

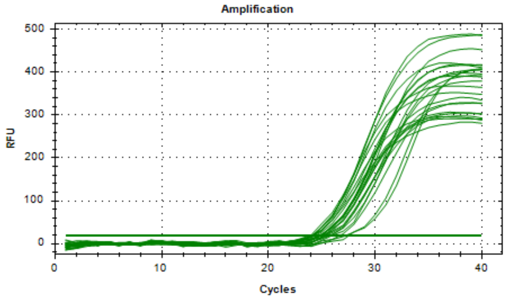

ATF6a amplification curve

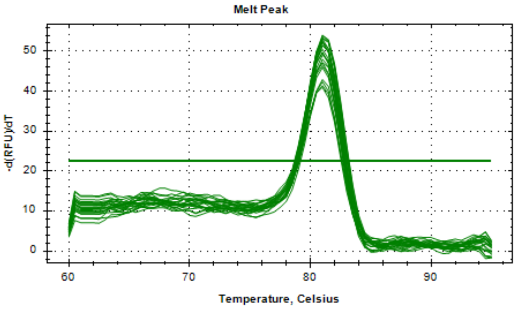

ATF6a solution curve

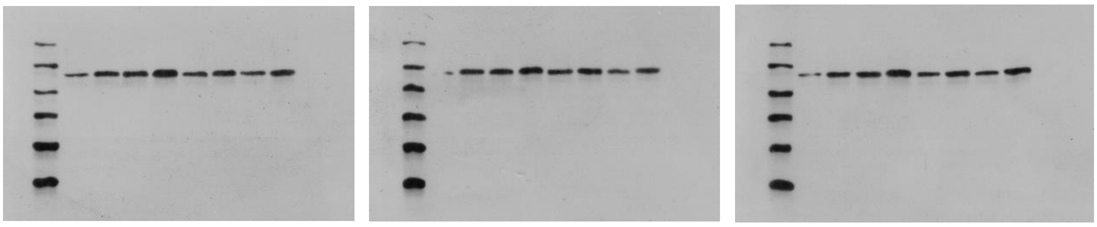

The Original blots/gels in turn are Control (Hcy-free), Hcy (100 μmol/L), Hcy+Si-NC, Hcy+Si-CTRP9, Hcy+Si-CTRP9+4-PBA (10 mmol/L, ERs inhibitor, MCE, HY-A0281), Hcy+GFP, Hcy+CTRP9, Hcy+CTRP9+TM (0.5 μg/mL, ERs agonist, MCE, HY-A0098) groups.

(C) CRP78 mRNA and protein expression levels.

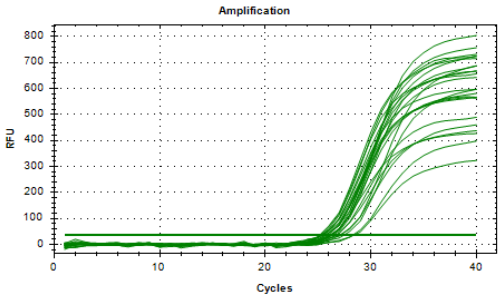

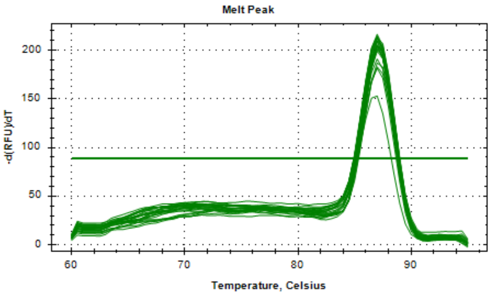

CRP78 amplification curve

CRP78 solution curve

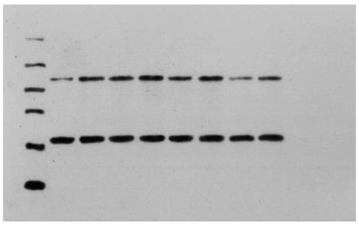

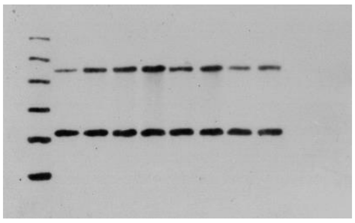

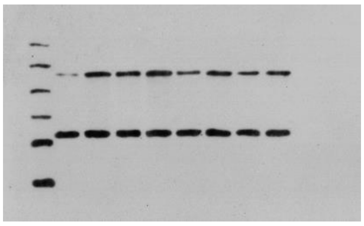

The Original blots/gels in turn are Control (Hcy-free), Hcy (100 μmol/L), Hcy+Si-NC, Hcy+Si-CTRP9, Hcy+Si-CTRP9+4-PBA (10 mmol/L, ERs inhibitor, MCE, HY-A0281), Hcy+GFP, Hcy+CTRP9, Hcy+CTRP9+TM (0.5 μg/mL, ERs agonist, MCE, HY-A0098) groups.

(D) Expression levels of IRE1a mRNA and p-IRE1a protein.

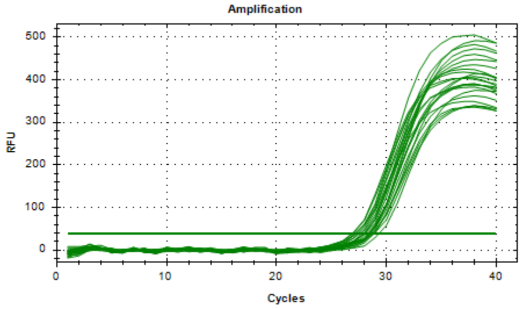

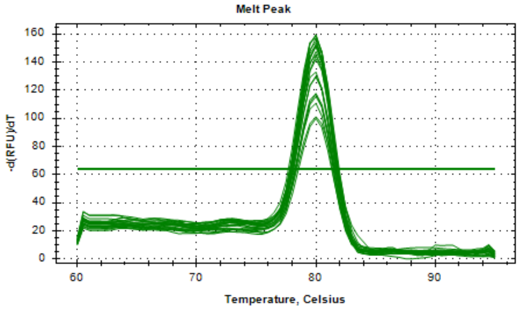

IRE1a amplification curve

IRE1a solution curve

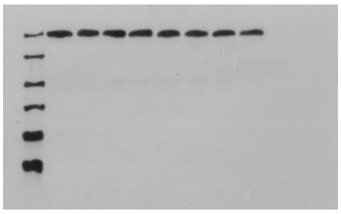

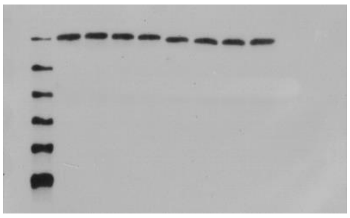

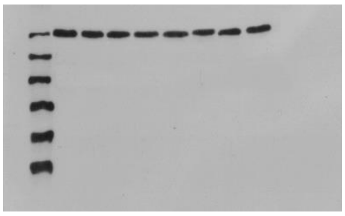

IRE1a protein expression in VSMCs.

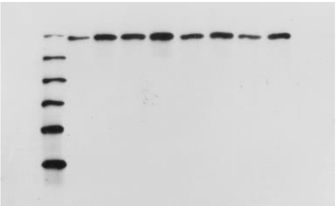

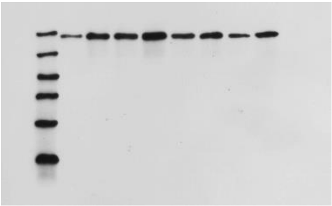

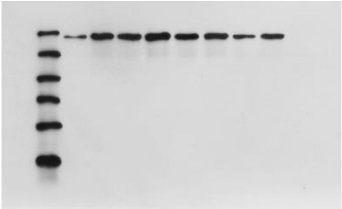

p-IRE1a protein expression in VSMCs.

The Original blots/gels in turn are Control (Hcy-free), Hcy (100 μmol/L), Hcy+Si-NC, Hcy+Si-CTRP9, Hcy+Si-CTRP9+4-PBA (10 mmol/L, ERs inhibitor, MCE, HY-A0281), Hcy+GFP, Hcy+CTRP9, Hcy+CTRP9+TM (0.5 μg/mL, ERs agonist, MCE, HY-A0098) groups.

(E) Expression levels of PERK mRNA and p-PERK protein.

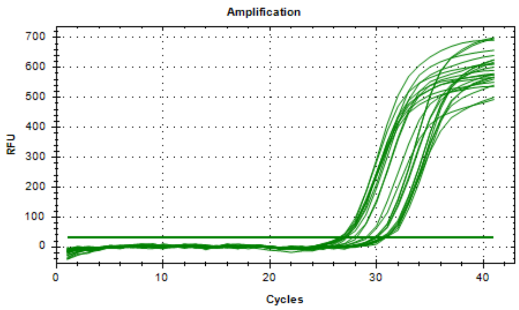

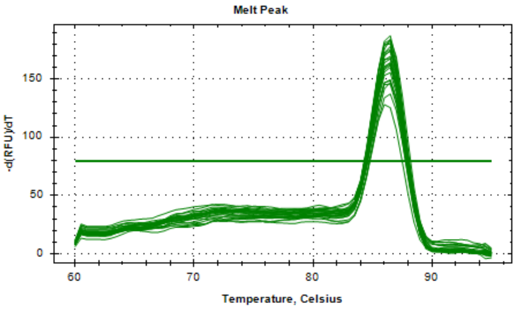

PERK amplification curve PERK solution curve

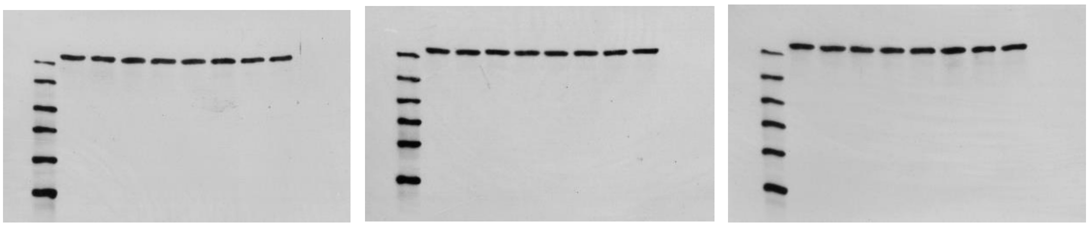

PERK protein expression in VSMCs.

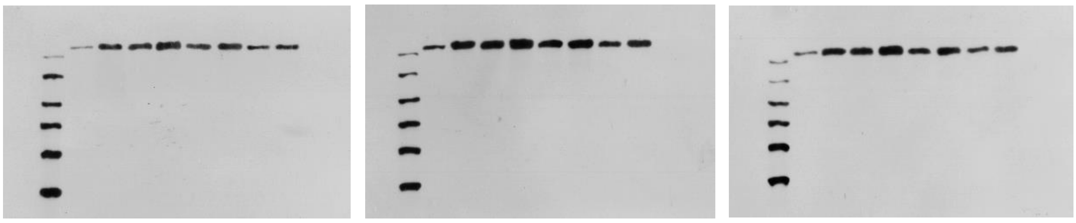

p-PERK protein expression in VSMCs.

The Original blots/gels in turn are Control (Hcy-free), Hcy (100 μmol/L), Hcy+Si-NC, Hcy+Si-CTRP9, Hcy+Si-CTRP9+4-PBA (10 mmol/L, ERs inhibitor, MCE, HY-A0281), Hcy+GFP, Hcy+CTRP9, Hcy+CTRP9+TM (0.5 μg/mL, ERs agonist, MCE, HY-A0098) groups.

(F) SREBP1c mRNA and protein expression levels.

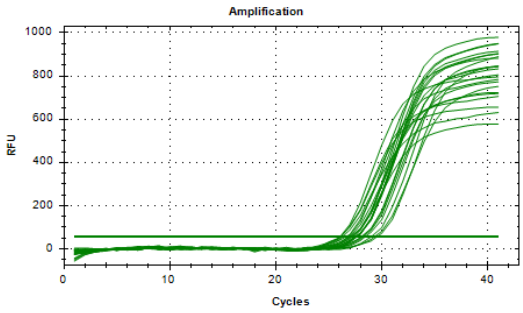

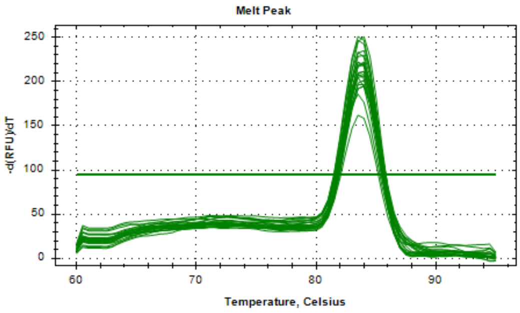

SREBP1c amplification curve SREBP1c solution curve

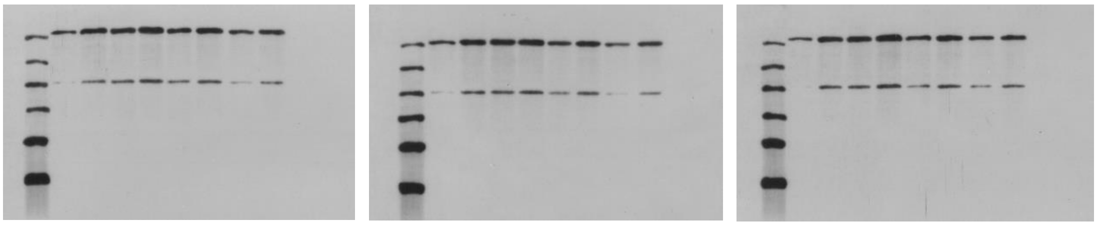

The Original blots/gels in turn are Control (Hcy-free), Hcy (100 μmol/L), Hcy+Si-NC, Hcy+Si-CTRP9, Hcy+Si-CTRP9+4-PBA (10 mmol/L, ERs inhibitor, MCE, HY-A0281), Hcy+GFP, Hcy+CTRP9, Hcy+CTRP9+TM (0.5 μg/mL, ERs agonist, MCE, HY-A0098) groups.

(G) SREBP2 mRNA and protein expression levels.

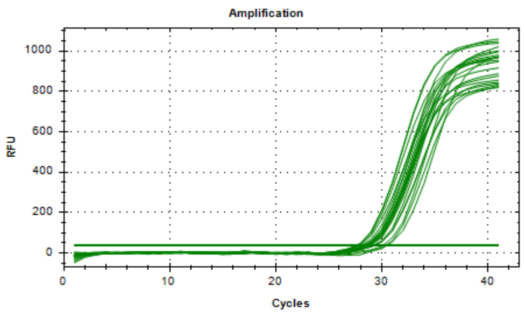

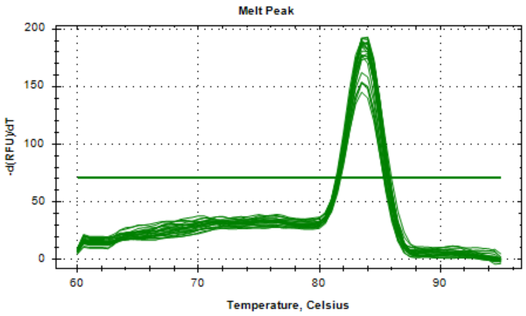

SREBP2 amplification curve SREBP2 solution curve

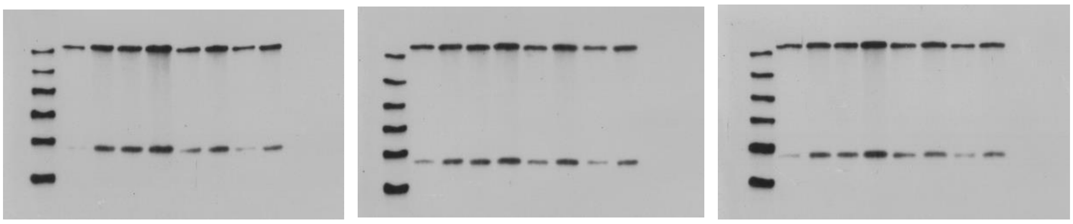

The Original blots/gels in turn are Control (Hcy-free), Hcy (100 μmol/L), Hcy+Si-NC, Hcy+Si-CTRP9, Hcy+Si-CTRP9+4-PBA (10 mmol/L, ERs inhibitor, MCE, HY-A0281), Hcy+GFP, Hcy+CTRP9, Hcy+CTRP9+TM (0.5 μg/mL, ERs agonist, MCE, HY-A0098) groups.

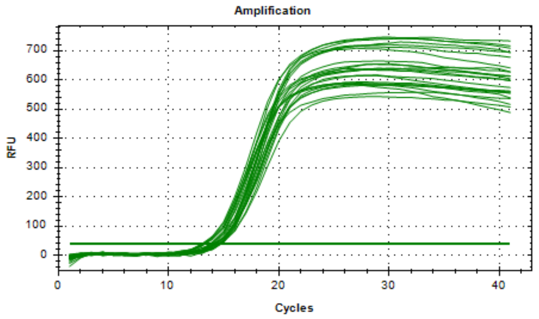

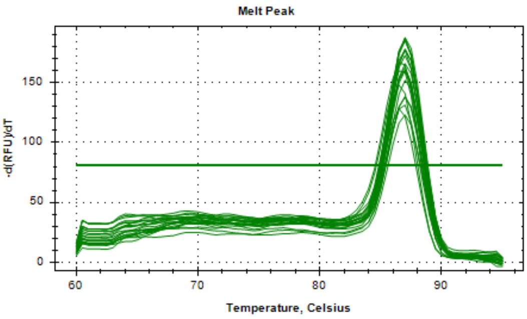

β-actin amplification curve β-actin solution curve

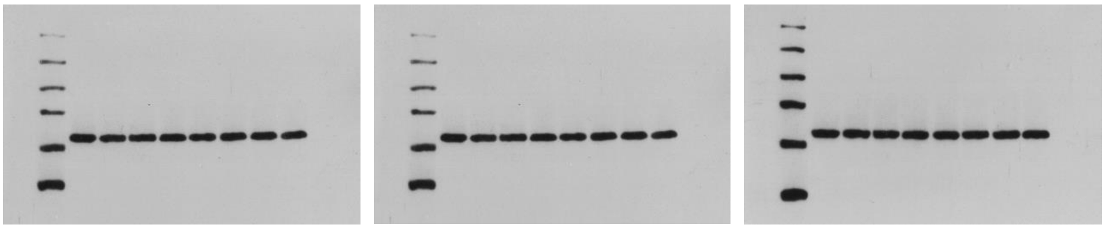

The Original blots/gels of β-actin.

Fig.5 Hypermethylation of CTRP9 promoter induced by Hcy via DNMT1

(D) Expression changes of CTRP9 and DNMT1 in VSMC of each group.

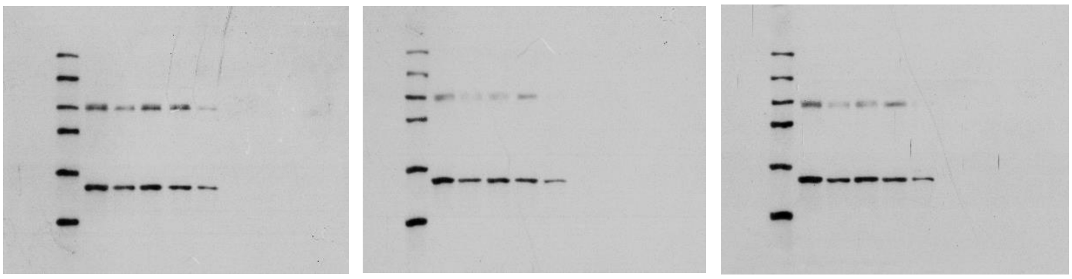

CTRP9 protein expression in VSMCs.

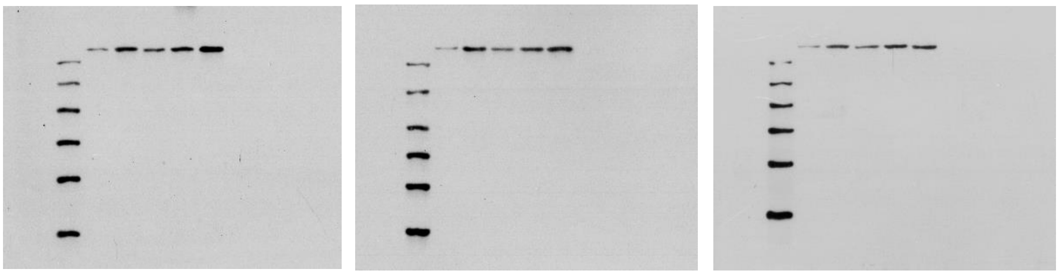

DNMT1 protein expression in VSMCs.

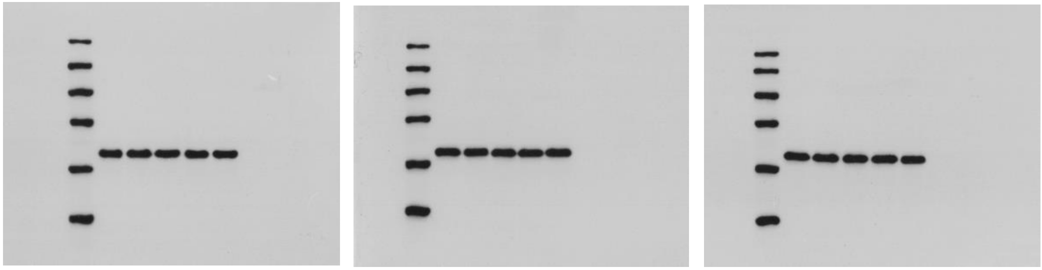

The Original blots/gels of β-actin.

The Original blots/gels in turn are Control, Hcy, Hcy+5-Azc (5 μmol/L, MCE, HY-10586), Hcy+Ad-GFP, and Hcy+Ad-DNMT1 groups.
